# *In Vitro* Ovicidal and Cestocidal Effects of Toxins from *Bacillus thuringiensis* on the Canine and Human Parasite *Dipylidium caninum*

**DOI:** 10.1155/2013/174619

**Published:** 2012-12-29

**Authors:** Guadalupe Peña, Fortino Agustín Aguilar Jiménez, Claudia Hallal-Calleros, Jorge Morales-Montor, Víctor Manuel Hernández-Velázquez, Fernando Iván Flores-Pérez

**Affiliations:** ^1^Centro de Investigaciones Biológicas, Universidad Autónoma del Estado de Morelos, Avenida Universidad 1001, Col. Chamilpa, 62209 Cuernavaca, MOR, Mexico; ^2^Facultad de Ciencias Agropecuarias, Universidad Autónoma del Estado de Morelos, Avenida Universidad 1001, Col. Chamilpa, 62209 Cuernavaca, MOR, Mexico; ^3^Facultad de Farmacia, Universidad Autónoma del Estado de Morelos, Avenida Universidad 1001, Col. Chamilpa, 62209 Cuernavaca, MOR, Mexico; ^4^Departamento de Inmunología, Instituto de Investigaciones Biomédicas, Universidad Nacional Autónoma de México, AP 70228, 04510 México, DF, Mexico; ^5^Centro de Investigación en Biotecnología, Universidad Autónoma del Estado de Morelos, Avenida Universidad 1001, Col. Chamilpa, 62209 Cuernavaca, MOR, Mexico

## Abstract

*Bacillus thuringiensis* is a gram-positive soil-dwelling bacterium that is commonly used as a biological pesticide. This bacterium may also be used for biological control of helminth parasites in domestic animals. In this study, we evaluated the possible ovicidal and cestocidal effects of a total protein extract of *B. thuringiensis* native strains on the zoonotic cestode parasite of dogs, *Dipylidium caninum* (*D. caninum*). Dose and time response curves were determined by coincubating *B. thuringiensis* proteins at concentration ranging from 100 to 1000 **μ**g/mL along with 4000 egg capsules of *D. caninum*. Egg viability was evaluated using the trypan blue exclusion test. The lethal concentration of toxins on eggs was 600 **μ**g/ml, and the best incubation time to produce this effect was 3 h. In the adult stage, the motility and the thickness of the tegument were used as indicators of damage. The motility was inhibited by 100% after 8 hours of culture compared to the control group, while the thickness of the cestode was reduced by 34%. Conclusively, proteins of the strain GP526 of *B. thuringiensis* directly act upon *D. caninum* showing ovicidal and cestocidal effects. Thus, *B. thuringiensis* is proposed as a potential biological control agent against this zoonosis.

## 1. Introduction 

*Bacillus thuringiensis* (*B. thuringiensis*) is a gram-positive bacterium occurring naturally in the soil and on plants. This bacterium produces proteins used for biological control against several agriculture pests and for some mosquito vectors of human diseases [1–3]. The proteins are classified into the crystal (Cry), and cytolytic (Cyt), vegetative insecticidal protein, and S-layers families; the mechanism of action of specific Cry proteins against agriculture pests has been characterized in detail [3]. *B. thuringiensis* has the advantage of being innocuous for humans, domestic animals and plants, and their proteins are highly biodegradable [4], making it a suitable and viable option to perform biological control on the parasites that infect mammals [2]. Certain proteins have been used in goats and ewes infected with *Haemonchus contortus, *resulting in effective control against larval and adult stages of this parasite [5, 6]. Furthermore, four strains of *B. thuringiensis*, referred to as GP123, GP138, GP130, and GP140, exhibited toxicity against *Rhipicephalus (Boophilus) microplus, *an ectoparasite that affects cattle [7]. It was also observed that the Cry5B protein of *B. thuringiensis* is effective *in vitro* and *in vivo* against the human nematode *Ancylostoma ceylanicum*. The effect was achieved by induction of a reduction in the number of eggs that is produced by the adult female and also decreased the development of the larval stage of the worm [8]. In a related study, the same protein administered in mice had antihelminthic activity against *Heligmosomoides bakeri *[9]. To date, there are no previous reports on the use of *B. thuringiensis* against cestodes. However, based on the described antihelminthic activity and its safety features, we propose in this study the bacterium's possible use against dipylidiasis, the parasitic disease caused by the cestode *D. caninum*, which is a zoonotic disease of public health importance that involve fleas as intermediate hosts and both humans and dogs as carriers of the adult parasite [10, 11]. The frequency of *D. caninum* in dogs worldwide varies from 0.1 to 44% [12–14]. In Mexico, higher frequencies, ranging from 54.7 to 60% have been reported [15–17]. This disease in dogs has been traditionally treated with drugs intended primarily to kill tapeworms, for example, praziquantel, pyrantel, and oxantel [18], however, there are parasites that had developed resistance to a number of these drugs [19]. Furthermore, these drugs may induce side effects, such as vomiting, nausea, headache, and hepatomegaly [20]. Particularly in the case of praziquantel, genotoxic potential has been established [21], which suggests the need to find other effective drugs that are safe and inexpensive. Taking into consideration this information, the aim of the present study was to explore the role of *B. thuringiensis* toxins against the adult cestode and egg capsules of *D. caninum* evaluating its *in vitro* effects on eggs development and survival and adult worm tegument thickness, which are key processes in the maintenance of the infectious cycle in dogs and humans. The *in vitro* effect of *B. thuringiensis* toxins on *D. caninum* was studied through pharmacological (dose and time effect) and microscopical (morphological studies) approaches to define the mechanisms of *B. thuringiensis* toxins' actions in the parasite. Our results demonstrated the ovicidal and cestocidal effects of *B. thuringiensis* toxins and promise a new therapeutic agent against *D. caninum*.

## 2. Materials and Methods

### 2.1. Parasites

Adult tapeworms of *D. caninum *were obtained from the intestine of infected dogs, which were humanely euthanized at the Canine Control Center in Tláhuac, México City. The method was previously evaluated by the University Animal Care and Use Committee to ensure compliance with international regulations and guidelines. After a lengthwise slitting, each intestine was inspected in search of the cestode in the lumen. The adult cestodes of *D. caninum* were identified based on the macroscopic appearance of proglottids [22]. The viability of parasites was determined by direct observation of the cestode under the microscope, counting as viable the parasites that exhibited full motion during 1 minute of observation [8].

### 2.2. Strain and Protein Recovery

The strain GP526 of *B. thuringiensis* used in this study belongs to the collection of the Vegetal Parasitology Laboratory, Center of Biological Research, University of Morelos, Mexico. The GP526 strain was isolated from a cyst of the phytoparasitic nematode *Meloidogyne* sp. (Tylenchida: Heteroderidae). This strain was grown using solid medium Luria-Bertani (LB) until complete sporulation (72 h at 28°C) and preserved in 60% of glycerol at 4°C until protein recovery. Crystal inclusions and spores were observed using an optical phase-contrast microscope and recovered using a bacteriological loop and suspended in sterile water. Protease inhibitor, (PMSF) 0.1 mM, was added to the suspension to avoid protein degradation [7], and total protein was quantified by the Bradford technique [23].

### 2.3. Egg Capsules Recovery

From each cestode, 2–4 gravid proglottids were chosen and analyzed using a microscope. The chosen proglottids were dissected, and the eggs were obtained and maintained at 4°C in 0.9% NaCl supplemented with antibiotics (penicillin and streptomycin, Sigma-Aldrich, St. Louis, MO, USA). The viability of eggs was measured according to Wang and cols. [24]. Briefly, egg hatching was induced using a 0.4% sodium hypochlorite solution. Viability was evaluated microscopically in eggs using the trypan blue exclusion test. 

### 2.4. Determination of LD50 y LT50 on Eggs

To calculate the median lethal dose (LD50) and median lethal time (LT50), dose-response and time-response curves were performed using concentrations of GP526 total protein. Each test was performed in quadruplicate and used 1000 eggs/mL in a final volume of 4 mL of culture medium. The ovicidal effect of the protein was quantified at intervals of 30 minutes during a 4-hour period and analyzed morphologically for the integrity of capsule eggs and viability using an optical microscope (40X objective lens). This analysis was performed by evaluating 10 microscope fields.

### 2.5. Determination of LD50 and LT50 on Adult Parasites

Six concentrations of GP526 protein, in the range of 0.25 to 10 mg/mL, were evaluated. For each of the concentrations tested we used a petri dish containing 15 mL of RPMI 1640 and 42 cestodes incubated at 37°C. A control group was administered with distilled water and PMSF (vehicle), and another with the commercial multispectrum intestinal wormer drug Pfizer Canex (oxantel embonate 543 mg/pyrantel embonate 143 mg/praziquantel 50 mg). The viability was determined by direct microscopic observation of parasite motility prior to the treatments and after the different times or doses of incubation. Each test was carried out in triplicate.

### 2.6. Morphological Study

Segments of viable adult *D. caninum* were obtained and immediately fixed in 10% paraformaldehyde during 72 h at 4°C [25]. The fixed segments were embedded in paraffin, and semiserial histological sections from 6 to 7 *μ*m were obtained. Sections were stained with hematoxylin-eosin to observe the general histological structure and measure the thickness of the integument. Ten microscope fields utilizing a 40X objective lens were evaluated in each section, performing 5 measurements of the tegument thickness for each of the fields and photodocumenting the teguments with an image analyzer [26]. 

### 2.7. Statistical Analysis

Concentration-response and time-response curves were estimated from six independent experiments; each experiment was performed with 1000 eggs/mL and 42 adults, freshly extracted from infected donor dogs. Each experiment was replicated in 24 different wells. The response variable used in statistical analysis was the sum of morphological integrity of capsule eggs and viability in the 24 wells with each treatment and time of exposure of the experiments. Data from the six replications of each experiment were pooled and expressed as the mean ± S.D. Data were analyzed using either Student's *t*-test or one-way ANOVA and subsequently with Dunnett's Multiple Comparison Test, depending on the experimental design. The motility data were analyzed using a one-way ANOVA test followed by a Tukey-Kramer test. Data concerning tegument thickness was analyzed using a nonparametrical ANOVA and Dunn's test. Differences were considered to be statistically significant with *P* < 0.05.

## 3. Results 

**Figure 1:**
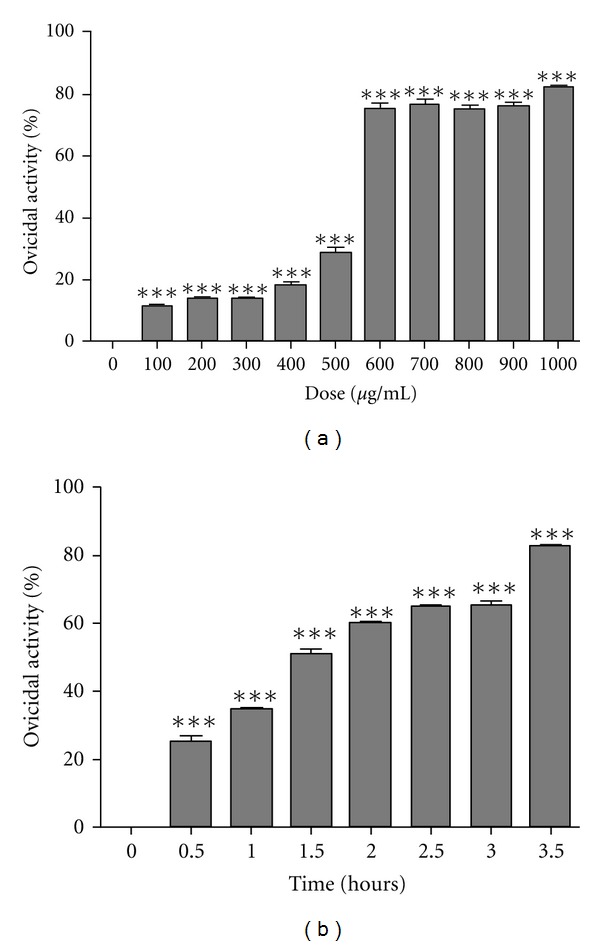
*B. thuringiensis* induces ovicidal effect on *D. caninum*. The ovicidal effect of *B. thuringiensis* is observed in a concentration-independent pattern (a) and maintained over time (b). In dose-response curves (panel (a)), *D. caninum* eggs treated with vehicle are referred as concentration zero. Data are represented as the mean ± SD. ***P < 0.05 compared to control.

**Figure 2:**
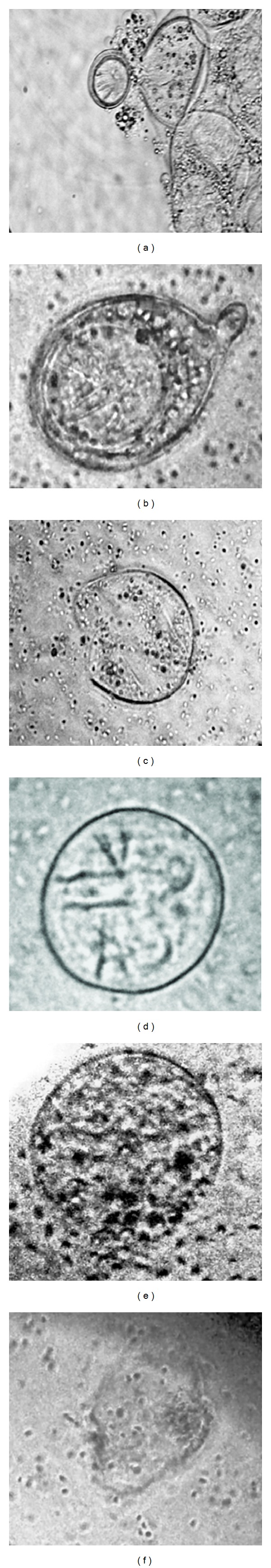
Lethal effect of the GP526 protein of *B. thuringiensis* on eggs of *D. caninum* (a). Lysis of the ovigerous capsule. (b) Fracture of the egg shell. (d) Exit of the exacant embryo. ((e) and (f)) Initial and final phases of the destruction of the exacant embryo.

A dose- and time-dependent ovicidal effect of GP526 total protein was found, as shown in Figure 1. A clear 75% ovicidal effect is observed at 600 *μ*g/mL of protein after 3.5 h after incubation. A higher dose (800 *μ*g/mL), showed the same effect (75.5%) at 3.5 h, and the optimal effectiveness under our experimental conditions was of 82.75% using 1000 *μ*g/mL of protein after 3.5 h of culture. In Figure 2, a panel of micrographs indicates morphological changes induced by *in vitro* treatment of eggs with 600 *μ*g/mL of GP526 protein extract after 3.5 hours. The protein is capable of lysing the ovigerous capsule, the composition of which is enriched with polysaccharides and glycoproteins; next, lysis of the egg surface containing the exacant embryo is observed, inducing hatching and subsequently causing their lysis and dissolution.

To determine the effect of the GP526 proteins in the adult worms, we tested random doses of 0.25, 0.5, 1.0, 1.5, 2.0, and 10.0 mg/mL on the motility of the parasite, and to determine if proteins of the bacteria were more or equally effective than commercial drugs, we used the commercial drug oxantel/pirantel/praziquantel as a positive control. We observed a 12% of reduction in motility in *D. caninum* using 0.25 mg/mL of GP526, and this percentage was decreased up to zero incubating with 10.0 mg/mL at 12 hours in culture (Table 1).

**Figure 3:**
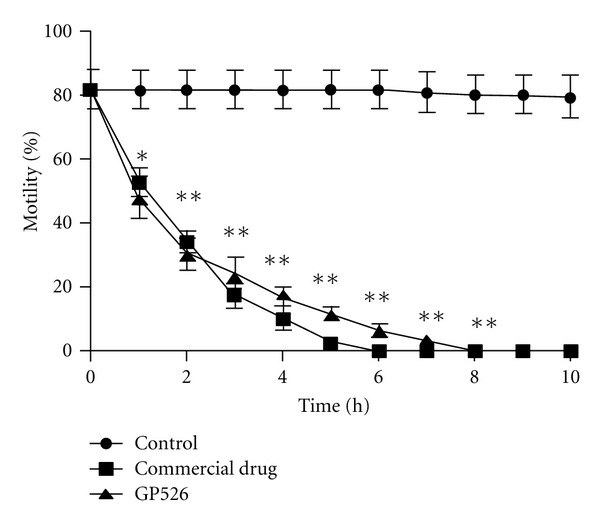
Total inhibition of motility in the adult cestode *D. caninum*. Total inhibition was reached by *in vitro* coincubation of the strain GP526 (10 mg/mL) of *B. thuringiensis* toxins and adult worms of *D. caninum*. *Significantly different from control group using a Tukey-Kramer test (*P < 0.01; **P < 0.001).

**Figure 4:**
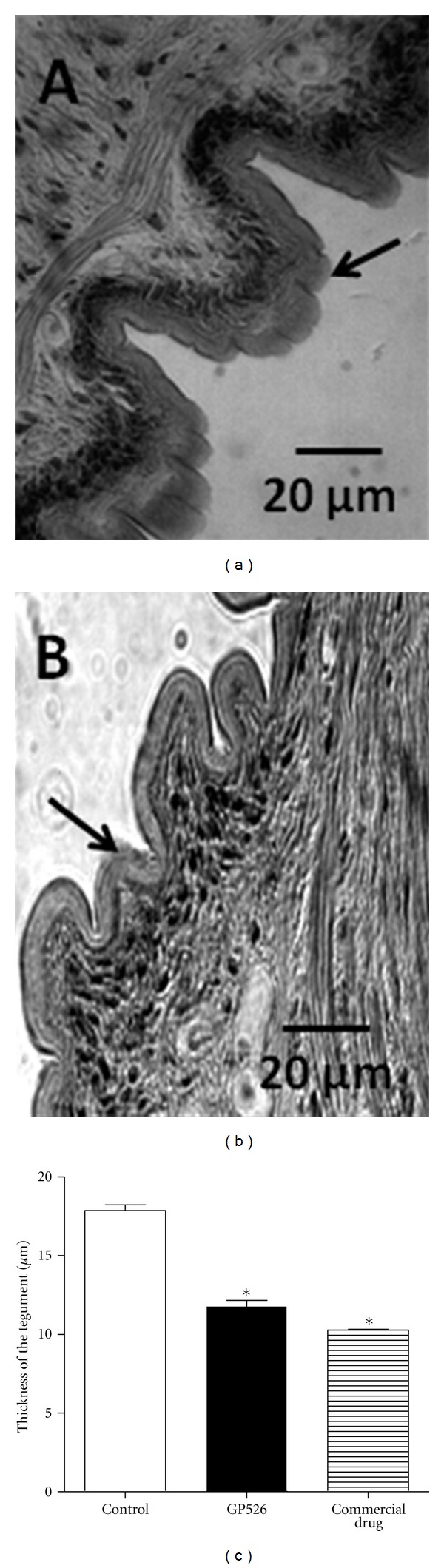
Reduction in the thickness of the tegument of *D. caninum* adult worms. Adult cestode untreated (a) or treated (b) with 10 mg for 8 hours post-incubation (40X). The strain GP526 of *B. thuringiensis* reduced the thickness tegument of the adult cestode *D. caninum* by 34% (c) (Dunn's test *P < 0.001).

In Figure 3, we show the time-dependent effect of 10.0 mg/mL of GP526 or the commercial drug on the adult cestode. In the control group treated with oxantel/pirantel/praziquantel, we observed that GP526 induced an inhibition of 50% in the mobility starting at the first hour of treatment, and this effect was increased up to 70% of inhibition after two hours, reaching a total inhibition after six hours of culture. Interestingly, a notably similar effect was observed with GP526 treatment. To determine the mechanisms by which GP526 affected the adult worm motility, we evaluated the possible tissue damage induced by *B. thuringiensis*, analyzing histological sections of *D. Caninum* adults. The thickness of the tegument was studied demonstrating that the strain GP526 was able to reduce by 34% the thickness of the adult cestode tegument (from 17.85 ± 0.35 to 11.79 ± 0.41) at 8 h after incubation with a concentration of 10 mg/mL (*P* < 0.001) (Figure 4). It is interesting to note that the effect of oxantel/pirantel/praziquantel was stronger because it inhibited the tegument thickness by 42% (from 17.85 ± 0.35 to 10.27 ± 0.22). 

## 4. Discussion 

In this study, we found an ovicidal and cestocidal effect of the strain GP526 of *B. thuringiensis* on *D. caninum*. It could be useful as a biological control method to interrupt the parasite's life cycle, destroying the egg and preventing the flea from becoming infected, thus mitigating the infection of dogs or humans. It should be noted that there is no commercial drug that has shown ovicidal effect on parasites, particularly in tapeworms. This aspect could also be important in preventing autoreinfection, and for this reason, considerable efforts are being undertaken in the search for drugs with ovicidal effects. Previous studies using the fungus *Pochonia* c*hlamydosporia,* report that after 15 days of *in vitro* after incubation on *D. caninum* eggs, the fungus affected only 49.2% of egg survival [27]. However, using the *B. thuringiensis* GP526 strain, eggs were killed in a shorter period of time, showing greater effectiveness relative to the fungus. In the case of the fungus *P. chlamydosporia*, it has been proposed that the ovicidal effect is based on enzymatic activity, but with respect to *B. Thuringiensis,* the mechanism of action is unknown. Furthermore, we observed that the strain GP526 of *B. thuringiensis* inhibited 100% of the adult cestode motility *in vitro*. Although there are no reports of *B. thuringiensis* effectiveness against tapeworms, it has been reported that six species of nematodes were found to be susceptible to proteins of *B. thuringienis* with the result of decreased motility and growth [28]. In another nematode, such as *Haemonchus contortus*, which affects small ruminants, it has been possible to inhibit the motility after two days of incubation with a concentration of 110 *μ*g/mg of body weight [5]. This concentration ratio is lower than the one observed in this work, wherein 100% of the motility of the adult cestode of *D. caninum *was inhibited (10 mg/mL of strain GP526). However, in our study, the inhibition of motility is faster, being recorded in only 8 h after incubation. The 100% effectiveness to inhibit motility in this study can be attributed to the way in which the cestodes have to nourish [29], unlike the nematodes. Cestodes feed throughout the tegument, meaning that the absorption surface of the protein may be higher, and because of the higher dose absorbed, the lethal time is shorter. It is important to take into account that at 10 mg/mL, the strain GP526 inhibited the motility of the cestode in a similar way to that of the commercial drug (oxantel/pirantel/praziquantel).

Biological control is the conscious use of living beneficial organisms, called natural enemies, for the control of pests [30]. Virtually all helminths, ecto- and endoparasites, have some natural enemies. Managing these natural enemies can effectively control parasite helminths [31, 32]. Often, the use of commercial drugs or other practices can create resistance to the drug; however, natural enemies are more difficult to manage by the parasite. Often, some of the most effective natural enemies of an organism are those that have coevolved with it in its native habitat. Natural enemies of parasites, particularly worms, must be carefully screened under rigid conditions to be certain that (1) they will provide benefit in controlling the target parasite, (2) they will not become parasites themselves, and (3) they do not harbor their own natural enemies that might interfere with their effectiveness or that of other natural enemies. The use of certain microbial insecticides (such as those containing *Bacillus thuringiensis*) is an example of inundation [33–35]. *Bacillus thuringiensis* is commonly used for controlling European corn borer larvae [36]. There is no doubt that well-researched applications of natural enemies can be highly effective. This approach includes the use of microbial insecticides, as well as many specific uses of predators of parasites and parasitic insects. Most likely, the common thread that exists with “failures” in the use of biological control of parasitic worms is a lack of knowledge. This ignorance encompasses both a lack of research needed to make recommendations for successful implementation and the user's lack of knowledge regarding the biology of parasites, their natural enemies, and their environment, all of which are crucial to successful biological control. 

## 5. Conclusions

Taking into consideration our findings, *B. thuringiensis* may have a dual therapeutic effect: egg removal and inhibiting the motility of the adult cestode and the integrity of its tegument. Although the commercial drug shows a greater effect on the thinning of the tegument of *D. caninum,* the drug has no effect on the eggs. Furthermore, *B. thuringiensis* has the advantage of being harmless to the treated animal and does not have the adverse effects related to the trade drug [2], which makes *B. thuringiensis* a potential candidate for use as antihelminthic in carrier animals. Future studies will be needed *in vivo* and at the tegument level to explain the molecular mechanisms of action by which proteins of strain GP526 acts against the adult cestode and eggs of *D. caninum*. Consideration of the biological and ecological needs of natural enemies is critical for the success of any biological control effort. While there are innumerable practices in the production system that may benefit or harm the natural enemies that researchers are seeking to manage, understanding the biology and life cycles of the desired species to conserve is the first step to achieving the best results.

## Figures and Tables

**Table 1:** Percentage of motility in *D. caninum *after incubation with different dose of GP26 strain proteins.

Experimental group	Time after incubation	
	12 hours	18 hours
Control (vehicle)	99.2 ± 0.80	98.4 ± 0.80
0.25 mg/mL GP526	88.13 ± 4.12	88.13 ± 4.12
0.5 mg/mL GP526	88.13 ± 4.12	88.13 ± 4.12
1 mg/mL GP526	85 ± 0.80	81.8 ± 2.11
1.5 mg/mL GP526	81.8 ± 2.11*	81.8 ± 2.11*
2.0 mg/mL GP526	77.0 ± 2.09**	77.0 ± 2.09**
10.0 mg/mL GP526	0**	0**
Oxantel/Pirantel/Praziquantel	0**	0**

Significant differences among groups were assessed by Tukey-Kramer test (*P < 0.01; **P < 0.001). Three repetitions were performed using a total of 126 adult worms per group.
